# Is Dark Triad Related to Regulatory Focus on Sexuality?

**DOI:** 10.1007/s10508-026-03513-2

**Published:** 2026-07-09

**Authors:** Andrés Barrios, Moisés R. Mebarak, Duban Romero, Martha Martinez, David L. Rodrigues

**Affiliations:** 1 Department of Psychology, Universidad del Norte, Barranquilla, Colombia; 2 Department of Psychology, Universidad Sergio Arboleda, Kra 46 KM5, Vía Puerto Colombia, 080001 Barranquilla, Colombia; 3 Department of Psychology, Universidad Simón Bolívar, Barranquilla, Colombia; 4 CIS-Iscte, Iscte-Instituto Universitário de Lisboa, Lisbon, Portugal

**Keywords:** Regulatory focus, Dark Triad, Narcissism, Psychopathy, Machiavellianism, Sex differences

## Abstract

Personality traits play an important role in shaping how individuals approach their sexual goals and regulate their sexual behavior. The present study (819 participants from Colombia) examined the association between different Dark Triad traits (i.e., Machiavellianism, narcissism, and psychopathy) and sexual regulatory focus, defined as a motivational orientation toward the pursuit of pleasure and rewards in sex (i.e., predominant focus on pleasure promotion) or the maintenance of safety and avoidance of negative health outcomes (i.e., predominant focus on disease prevention). We also explored whether these associations were moderated by gender. Results showed that all three Dark Triad traits were positively associated with promotion focus scores, and only psychopathy was negatively associated with prevention focus scores. Despite a priori gender differences in Dark Triad traits and promotion focus scores, we found no evidence of moderation by gender in any of the tested associations. Our findings suggest that Dark Triad traits shape individuals’ motives to pursue sexual pleasure or avoid sexual health threats, thus contributing to the literature on personality and sexuality. Theoretical and applied implications are discussed.

## Introduction

Personality traits play a central role in shaping how individuals perceive, approach, and regulate their sexual experiences (Allen & Walter, 2018; Kurpisz et al., 2016). Individual differences in personality have been linked not only to sexual interests and motivations in sex, but also to decision-making processes related to intimacy, risk-taking, and interpersonal boundaries (Fincham & May, 2017; Hatfield et al., 2011; Reinhardt & Reinhard, 2023). These associations are particularly relevant when examining personality traits, such as those encompassed by the Dark Triad (i.e., psychopathy, Machiavellianism, and narcissism), which have been consistently associated with distinctive interpersonal and sexual patterns (Jones & Paulhus, 2014; Paulhus & Williams, 2002; Rogoza & Cieciuch, 2020).

Dark Triad traits are characterized by specific dispositional tendencies. Psychopathy has been linked to impulsivity, sensation seeking, and reduced behavioral inhibition (Jones & Paulhus, 2014). Machiavellianism reflects a strategic and manipulative interpersonal style oriented toward personal gain (Rogoza & Cieciuch, 2020). Narcissism involves grandiose self-views, entitlement, and a strong need for admiration, which may shape sexual interactions through heightened self-focus and expectations of special treatment (Paulhus & Williams, 2002).

In the sexuality domain, a substantial body of research has documented associations between Dark Triad traits (either all or a subset) and harmful or problematic sexual behaviors, such as sexual coercion (Blinkhorn et al., 2015; Lyons et al., 2020; O’Connell & Marcus, 2016; Pavlović et al., 2019). Specifically, Machiavellianism and psychopathy have also been linked to a higher likelihood of engaging in non-consensual extradyadic sexual behaviors and intentions, often without perceiving such behaviors as violations of relationship norms (Brewer & Abell, 2015; Brewer et al., 2023), hostile interpersonal environments conducive to sexual harassment (Burns, 2019; Tenbrunsel et al., 2019; Zeigler-Hill et al., 2016), endorsement of myths surrounding sexual abuse (Jonason et al., 2017), and tendencies to blame victims of sexual assault (Brewer et al., 2021; Chung & Sheridan, 2021; Lyons et al., 2022). Dark Triad traits have also been associated with a larger number of sexual partners and more frequent casual or short-term relationships (Bonfá Araujo et al., 2022; Burtăverde et al., 2021a; Jonason et al., 2009; Jones & De Roos, 2017; Moore et al., 2020; Tsoukas & March, 2018; Valentova et al., 2020), lower levels of anxiety and fear in intimate situations, arguably indicating higher sexual confidence or perceived sexual quality (Pilch & Smolorz, 2019; Steininger & Pietschnig, 2022), or self-enhancing perceptions of attractiveness and value as sexual partners, which arguably shape mating success and interpersonal dynamics (Borráz León & Rantala, 2021; Csajbók et al., 2023; Leslie-Evans et al., 2022).

Building upon this evidence, we suggest that the Dark Triad can offer a useful framework to understand variability in the way individuals’ approach and pursue their sexual goals. Drawing from the regulatory focus theory (Higgins, 2015), individuals predominately focused on prevention are driven by safety maintenance, risk avoidance, and strive to minimize potentially negative outcomes that can result from their behavior (e.g., more likely to adhere and maintain medical prescriptions), whereas individuals predominately focused on promotion are driven by pleasure seeking, reward attainment, and strive to gain potentially positive outcomes from their behavior (e.g., more likely to gamble for money; see also Scholer et al., 2019). This framework has been extended to the field of sexual experiences and practices. Indeed, individuals with a predominant focus on prevention (vs. promotion) tend to perceive more sexual health threats, enact safer sex practices (e.g., report using condoms more often and having greater control over condom use with casual partners), score higher on sexual restraint, and report a more restricted sociosexuality (Rodrigues et al., 2019, 2020). In contrast, individuals with a predominant focus on promotion (vs. prevention) tend to be more extraverted, perceive condoms as pleasure barriers, report having condomless sex with a larger number of casual partners, and report experiencing more sexual pleasure (Evans-Paulson et al., 2022; Rodrigues & Lopes, 2023; Rodrigues et al., 2024a, 2024b; Romero et al., 2025).

Importantly, sexual regulatory focus differs conceptually from constructs such as sociosexual desire or hypersexuality, as it emphasizes how individuals regulate sexual goals and decisions rather than the intensity of sexual desire or the frequency of sexual behavior (Rodrigues et al., 2019). In this sense, regulatory focus reflects a motivational orientation toward prioritizing sexual health (i.e., disease prevention focus) or prioritizing sexual wellbeing (i.e., pleasure promotion focus; Rodrigues et al., 2020). As previous findings suggest that Dark Triad traits are associated with pleasure-seeking tendencies (Burtăverde et al., 2021a; Jonason et al., 2009; Moore et al., 2020), we argue for a similar strong association with pleasure promotion motives, whereas prevention focus may show weaker or negative associations, particularly for traits characterized by relaxing sexual criteria.

Despite extensive evidence linking Dark Triad traits to sexual attitudes and behaviors (Brewer & Abell, 2015; Burtăverde et al., 2021a, 2021b; Jonason et al., 2017; O’Connell & Marcus, 2016; Smith et al., 2019; Zeigler-Hill et al., 2016), little is known about how these traits relate to motivational self-regulation in sexuality, particularly with respect to promotion and prevention orientations. Previous studies have shown that sexual regulatory focus is associated with condom use, intentions to engage in safer sex practices, and perceived threats to sexual health (Rodrigues et al., 2019, 2021), yet its relationship with Dark Triad traits has not been directly examined. There have been findings regarding gender in this context. For example, men high on psychopathy have bigger intentions toward infidelity and sociosexual attitudes than women (Moore et al., 2020), that Dark Triad traits are strongly correlated to self-perceived attractiveness, and the acquisition of sexual partners in both men and women (Borráz-León & Rantala, 2021). Examining these associations may provide a more nuanced understanding of sexual decision-making processes in individuals with elevated Dark Triad traits and help clarify potential pathways linking personality, motivation, and sexual behavior. Accordingly, the present study aims to examine the relationships between the three Dark Triad traits and sexual regulatory focus, specifically prevention and promotion orientations.

## Method

### Participants and Procedure

Data were collected between September 2021 and May 2022 through an online survey hosted on the QuestionPro platform. The study targeted individuals at least 18 years old (*M* = 23.10, *SD* = 7.04), residing in Colombia, and who had engaged in sexual activity. Research assistants shared the survey link through a non-probabilistic snowball sampling method. Prospective participants, upon accessing the survey, were presented with ethical information, and obtaining informed consent was a prerequisite to proceeding with the study. The survey started with standard demographic information (e.g., gender, age, location, sexual orientation, education level, and marital status), followed by the primary measures and additional measures unrelated to the current investigation. Embedded in the survey, we also included an attention check item (e.g., “To verify that you are reading each question, please answer the 'Not at all satisfied' option”). At the end of the survey, participants were thanked and debriefed about the study’s objectives. A total of 1005 individuals assessed the online survey, of which 186 were removed due to missing or random responses. The final sample (*N* = 819) comprised 59.5% were women, 82% were heterosexuals, 55% had university education, and 54.5% were single at the time of participating in the study.

## Measures

### Dark Triad Traits

Participants completed the Spanish version (Nohales Nieto, 2015) of the Short Dark Triad (Jones & Paulhus, 2014), indicating their agreement (1 = *Disagree strongly to* 5 = *Agree strongly*) with 27 items assessing narcissism (9 items; e.g., “I insist on getting the respect I deserve”), psychopathy (9 items; e.g., “People who mess with me always regret it”), and Machiavellianism (9 items; e.g., “There are things you should hide from other people to preserve your reputation”). Responses were averaged for each subscale, with higher scores indicating more psychopathy (α = .72) and Machiavellianism (α = .72), but it was not the case for narcissism (α = .57).

#### Sexual Regulatory Focus

We used the Regulatory Focus in Sexuality scale (Rodrigues et al., 2019; Spanish version by Rodrigues et al., 2020; validated in Colombia by Romero et al., 2025) to assess prevention motives (three items; e.g., “Not being careful enough with my sex life has gotten me into trouble at times”) and promotion motives in sex (six items; e.g., “I often think about how I can achieve (or create) a successful sex life”). Responses to each item were given in 7-point response scales (1 = *Not at all true of me to* 7 = *Very true of me*), and mean scores were computed for the Prevention subscale (α = .73) and Promotion subscale (α = .81).

### Data Analysis

We started by computing overall correlations between all variables, gender differences between the variables using independent-samples *t*-tests with Welch’s correction (effect sizes were estimated using Cohen’s *d*), and correlations separate by gender. Then, we examined the unique contribution of each Dark Triad trait on sexual regulatory focus motives by estimating two multiple linear regression models—one with pleasure promotion motives as the outcome and one with disease prevention motives as the outcome. In both models, Machiavellianism, narcissism, and psychopathy were entered simultaneously as predictors, alongside their respective interactions with gender. When interactions with gender reached significance, we followed up with simple slope analyses.

## Results

Statistical analyses were used to compare the sexual regulatory focus in men and women. The results showed that males are slightly more focused on sexual promotion than females, *p* = .004, and that there are no differences in sexual prevention according to gender (*p* = .488). Also, when scores on Machiavellianism, narcissism, and psychopathy were compared, it was found that males have higher Machiavellianism (*p* < .001), higher narcissism (*p* = .002), and higher psychopathy (*p* < .001) than females (see Table 1).

**Table 1:** Gender differences

	*t *(df)	*d*	Female M (SD)	Male M (SD)
Prevention	.69 (726.62)	.049	67.98 (27.32)	66.65 (27.64)
Promotion	− 2.85 (788.86)^***^	.198	59.88 (24.82)	64.50 (21.87)
SD3-Machiavellianism	− 5.66 (710.24)^***^	.399	44.73 (14.80)	50.76 (15.48)
SD3-Narcissism	− 3.05 (702.17)^***^	.215	50.48 (11.79)	53.09 (12.53)
SD3-Psychopathy	− 7.87 (697.98)^***^	.556	26.34 (14.05)	34.43 (15.06)

^***^p < .001, ^**^p < .01, ^*^p < .05

All scores were transformed to a 0–100 range to facilitate comparability across scales [(observed − minimum) / (maximum − minimum) × 100]

When examining the associations between the Dark Triad and regulatory focus by gender, both clear similarities and some specific differences emerge. In both groups, Dark Triad traits were negatively associated with prevention focus and positively associated with promotion focus, indicating a common overall pattern for women and men. However, the strength and statistical significance of some correlations differed by gender. Among women, prevention focus had a significant negative correlation to Machiavellianism (*r* =  − .24, *p* < .001) and psychopathy (*r* =  − .32, *p* < .001), but not to Narcissism (*r* =  − .05, ns), whereas in men all three negative associations were significant, although weaker for Narcissism (r =  − .111, *p* < .05). Regarding promotion focus, positive correlations with Machiavellianism, narcissism, and psychopathy were significant and of similar magnitude in both genders, with slightly higher coefficients for men in Machiavellianism and psychopathy and virtually equivalent values for narcissism (see Table 2).

**Table 2:** Correlations for women and men

	1	2	3	4	5
1. SD3-Machiavellianism	–	0.28***	0.47***	− 0.20***	0.21***
2. SD3-Narcissism	0.21***	–	0.22***	− 0.11*	0.25***
3. SD3-Psychopathy	0.52***	0.13**	–	− 0.28***	0.22***
4. Disease prevention motives	− 0.24***	− 0.05	− 0.32***	–	− 0.33***
5. Pleasure promotion motives	0.19***	0.24***	0.15***	− 0.30***	–

Values below the diagonal indicate correlations for the female group, and values above the diagonal indicate correlations for male group. ***p < .001, **p < .01, *p < .05

Next, Machiavellianism, narcissism, and psychopathy were included as predictors of sexual promotion. According to results, positive relationships were detected for narcissism (*p* < .001), Machiavellianism (*p* = .006), and psychopathy (*p* = .021), with narcissism showing the strongest effect. On the other hand, no statistical evidence was found linking gender and age to sexual promotion (all *p* > .05). In the case of prevention focus, negative effects of Machiavellianism (*p* = .011) and psychopathy (*p* < .001) on sexual promotion were detected (see Table 2). Additionally, being male did not have a significant effect on prevention focus (*p* = .05). Also, narcissism (*p* = .66) and age (*p* = .20) did not show significant effects (Table 3).

**Table 3:** Regression model for regulatory focus

	Promotion		Prevention	
	B (SE)	*t*	B (SE)	*t*
Intercept	31.26 (4.57)	6.83^***^	86.33 (5.27)	16.39^***^
Age	−.13 (.11)	− 1.18	.16 (.12)	1.26
Male	1.34 (1.65)	.81	3.73 (1.90)	1.96
SD3-Narcissism	.40 (.07)	6.03^***^	−.03 (.08)	−.43
SD3-Machiavellianism	.17 (.06)	2.75^**^	−.18 (.07)	− 2.54^*^
SD3-Psychopathy	.15 (.06)	2.30^*^	−.47 (.07)	− 6.48^***^

^***^p < .001, **p < .01, *p < .05

## Discussion

In this study, we explored whether Dark Triad traits—Machiavellianism, narcissism, and psychopathy—were associated with sexual regulatory focus in a sample of women and men in Colombia. Overall, our findings suggest that Dark Triad traits shape how individuals self-regulate and approach their sexual health and wellbeing goals, with the strength and direction of such associations varying for traits and regulatory motives.

We found significant positive associations between all Dark Triad traits and pleasure promotion motives. This pattern is consistent with previous literature showing that individuals with higher levels of Dark Triad traits tend to report a larger number of sexual partners, engage more in casual or short-term relationships, and be more sociosexually unrestricted (Bonfá-Araujo et al., 2022; Del Río et al., 2019; Jonason et al., 2009; Jones & De Roos, 2017; Moore et al., 2020; Tsoukas & March, 2018; Valentova et al., 2020). Because sexual regulatory focus is framed as a motivational system through which sexual decisions are guided (Rodrigues et al., 2019), our results suggest that individuals with higher levels of Dark Triad traits are more inclined toward pleasure maximization and reward-seeking goals in sex, instead of health safety. Several characteristics commonly associated with Dark Triad traits may help contextualize this tendency. Prior research has shown that Dark Triad traits are linked with lower sexual disgust sensitivity, fewer perceived sexual boundaries, and greater tolerance of taboo or socially disapproved sexual behaviors and attitudes (Burtăverde et al., 2021a; Stefanczyk et al., 2023). In addition, individuals higher in Dark Triad traits report a greater prevalence of sexual fantasies involving unconventional or socially frowned upon themes and a higher cognitive preoccupation with sexual relationships (Baughman et al., 2014; Brown et al., 2020; Burtăverde et al., 2021b; Müezzin & Okray, 2021; Pilch & Smolorz, 2019; Steininger & Pietschnig, 2022). Together, these tendencies align with a promotion-focused orientation characterized by lower sexual restraint and greater openness to sexual experiences, even in the presence of potential risks (Rodrigues et al., 2019, 2021).

Among the three traits, narcissism emerged as the strongest predictor of pleasure promotion motives. This finding is consistent with evidence suggesting that narcissistic individuals experience fewer negative emotions in intimate contexts, perceive themselves as more attractive and desirable sexual partners, and show heightened sexual assertiveness and self-focus (Borráz-León & Rantala, 2021; Csajbók et al., 2023; Leslie-Evans et al., 2022; Pilch & Smolorz, 2019; Steininger & Pietschnig, 2022). Based on our findings, then, these individuals are also driven to seek rewarding and affirming sexual experiences.

In contrast, we found a significant negative association between psychopathy and disease prevention motives. Aligned with this, previous research has linked psychopathy, and more particularly its impulsive component, to unrestricted sociosexuality, more involvement in short-term sexual encounters, and riskier sexual behaviors, including less frequent condom use (Baughman et al., 2014; Burtăverde et al., 2021a, 2021b; Freyth & Jonason, 2023; Jones & De Roos, 2017; Lyons et al., 2020; Moore et al., 2020). From a sexual regulatory focus framework (Rodrigues et al., 2019), then, our results suggest that individuals who score higher on psychopathy are less inclined toward health safety, sexual restraint, or long-term consequences. Machiavellianism showed weaker and more complex associations with both regulatory motives, having smaller significant positive associations with pleasure promotion motives and smaller significant negative associations with disease prevention motives. This pattern may reflect the strategic and self-interested nature of Machiavellian traits. On the one hand, Machiavellianism has been linked to higher sexual motivation, infidelity, sexual deception strategies, and maladaptive sexting behaviors (Brewer & Abell, 2015; Brewer et al., 2016, 2019; Morelli et al., 2021; Smith et al., 2019). On the other hand, Machiavellian individuals are typically described as cautious, reputation conscious, and oriented toward long-term self-interest, which may discourage overtly reckless or socially damaging sexual behavior (Jones & Paulhus, 2014; Rogoza & Cieciuch, 2020). From this perspective, Machiavellianism may function as a flexible regulatory style that can align with either promotion or prevention depending on contextual incentives. Future research including sociosexuality and sexual motivation measures may help clarify this dual pattern.

Regarding gender, although previous studies have documented gender differences in several sexuality related outcomes associated with Dark Triad traits (Brewer et al., 2016; Brewer et al., 2019; Brewer et al., 2021; Del Río et al., 2019; Freyth & Jonason, 2023; Lyons et al., 2020; O'Connell & Marcus, 2016; Zeigler-Hill et al., 2016), in this study small mean differences in regulatory focus were observed between men and women, but these differences did not remain significant once Dark Triad traits were included in the regression models. This indicates that motivational orientations toward sexual promotion or prevention may operate similarly across genders when personality characteristics are considered.

An additional contribution of the present study lies in its focus on a Latin American sample, a context that remains underrepresented in empirical research on personality and sexuality. Cultural norms in many Latin American societies often involve stronger social regulation of sexual behavior, more traditional gender role expectations, and higher levels of moral and social sanctioning surrounding sexuality, which may influence how individuals negotiate pleasure, restraint, and risk in intimate contexts (Abdo, 2020; Bozon et al., 2009). Within such settings, sexual regulatory focus may become particularly salient, as individuals navigate tensions between personal motivations and socially prescribed norms. Examining how Dark Triad traits relate to sexual regulatory focus in this cultural context helps extend existing findings beyond predominantly Western, educated, and industrialized samples, and provides a more culturally informed understanding of sexual self-regulation. Future research should further explore cross cultural comparisons to clarify the extent to which these relationships are culturally invariant or context dependent.

Notably, the findings herein reported should be interpreted considering some limitations. The use of a non-probabilistic sample composed primarily of young and highly educated individuals limits the generalizability of the results; likewise, the low reliability of the narcissism subscale may introduce noise measurement in this construct. Therefore, it is suggested that this study be replicated using alternative instruments that assess the Dark Triad. Likewise, future studies should replicate these findings in more diverse samples and incorporate additional variables such as sociosexuality, sexual motivation, and relational context. Such efforts may further elucidate how personality traits shape sexual self-regulation in both adaptive and maladaptive ways.

### Conclusion

The present study offers novel evidence that Dark Triad personality traits are meaningfully associated with sexual regulatory focus. Overall, higher levels of Machiavellianism, narcissism, and psychopathy were linked to a greater promotion-focused orientation toward sexuality, whereas psychopathy was additionally associated with lower prevention focus. These findings suggest that personality traits characterized by impulsivity, self-enhancement, and strategic self-interest may shape sexual self-regulation by prioritizing pleasure and reward over safety and restraint. Importantly, the associations between Dark Triad traits and regulatory focus were largely consistent across genders, indicating that motivational orientations toward sexual promotion and prevention are better explained by personality characteristics than by gender alone. By situating regulatory focus within the broader literature on personality and sexuality, this study contributes to a more nuanced understanding of how individual differences in personality relate to sexual self-regulation, while highlighting the need for future research using diverse samples and additional motivational and behavioral indicators.

## Data Availability

Data are available at https://osf.io/49kte/.
